# Antibiotic Consumption on Dairy and Beef Cattle Farms of Central Italy Based on Paper Registers

**DOI:** 10.3390/antibiotics9050273

**Published:** 2020-05-25

**Authors:** Laura Ferroni, Carmela Lovito, Eleonora Scoccia, Gastone Dalmonte, Marta Sargenti, Giovanni Pezzotti, Carmen Maresca, Claudio Forte, Chiara Francesca Magistrali

**Affiliations:** 1 Istituto Zooprofilattico Sperimentale dell’Umbria e delle Marche “Togo Rosati”, 06121 Perugia, Italy; l.ferroni@izsum.it (L.F.); Carmela.Lovito@izsto.it (C.L.); e.scoccia@izsum.it (E.S.); g.dalmonte@izsum.it (G.D.); m.sargenti@izsum.it (M.S.); g.pezzotti@izsum.it (G.P.); c.maresca@izsum.it (C.M.); c.magistrali@izsum.it (C.F.M.); 2 Istituto Zooprofilattico Sperimentale del Piemonte, Liguria e Valle d’Aosta, 10154 Torino, Italy

**Keywords:** antibiotic consumption (AMC), dairy cattle, beef cattle, DDD_vet_, DCD_vet_, antimicrobial resistance, antibiotic use

## Abstract

The overuse of antibiotics in livestock contributes to the antibiotic resistance pandemic. The assessment of the actual antibiotic consumption is crucial in limiting the expansion of the problem effectively. The aim of this study was to provide the first qualitative and quantitative analysis of antimicrobial usage using data from paper-based registers on dairy and beef farms located in the Umbria region, Italy. Antimicrobial therapies of a one-year period were collected from 101 farms with at least 50 cattle each. Defined daily doses (DDD_vet_) and defined course doses (DCD_vet_) were calculated per administration route and antimicrobial class. The total courses administered were fewer in beef (330.7 × 10^−3^ DCD_vet_/year) than in dairy farms (1034.1 × 10^−3^ DCD_vet_/year). The use of the highest priority critically important antimicrobials (HPCIAs) was higher (*p* = 0.0033) in dairy than in beef herds. In terms of DDD_vet_, the parenteral fluoroquinolone administration ranked second and fourth on dairy and beef farms, respectively; the consumption of beta-lactams was ten times higher on dairy than on beef farms. Our results confirm that intensive dairy management practices are associated with increased antibiotic consumption and highlight the necessity to strengthen the existing stewardship programs by involving all stakeholders in effective antimicrobial resistance reduction plans.

## 1. Introduction

The pandemic of antibiotic resistance (AMR) poses a global threat for human health. Human-to human transmission of antibiotic-resistant bacteria is the main way of acquiring and spreading AMR; however, other ecosystems also contribute to its emergence and dissemination. Among them, livestock is considered to play an important role [1,2,3]. The overuse of antibiotics in farm animals is a major contributor to AMR; antibiotics put selective pressure on microbial populations and result in the spread of resistant bacteria [3,4]. Therefore, stewardship programs aimed at reducing the consumption of antibiotics by farm animals have been included in action plans against AMR [3,5,6]. In most of these plans, surveillance is based on the systematic collection of antibiotic consumption data, which constitute an invaluable tool for the analysis of the progress of antimicrobial stewardship programs either at the farm, regional, national, or supranational levels [2,5,7].

In Europe, the European Medicines Agency (EMA) has been monitoring antibiotic consumption in animal production since 2010, through the European Surveillance of Veterinary Antimicrobial Consumption (ESVAC) project. The project has collected data on antibiotic sales and an open-access database and yearly reports have been provided to the public since 2011. The unit of measurement is a result of the weight of the active substance sold in each country, expressed in mg, adjusted by the animal population of the same country (population correction unit (PCU)). According to the early ESVAC reports, Italy was among the European countries with the highest antibiotic consumption in agriculture [8]. Despite an overall reduction in antibiotic use, Italy, with its 273.8 mg/PCU, is well above the overall European consumption, 107.0 mg/PCU (maximum: 423.1 mg/PCU, minimum: 3.1 mg/PCU), maintaining its unenviable second place position in 2017 [8].

The analysis of national sales data has made the identification of antibiotic consumption trends possible. Even more importantly, a correlation between antibiotic consumption and antibiotic resistance at the country level has been shown [2]. However, intercountry comparisons should be interpreted with caution, owing to differences in animal populations and farming systems. A limitation of the system is that it does not take the efficacy of different drugs into account, as its unit of measurement is mg. Moreover, the attribution of antibiotic consumption to a given species is only presumptive, as many products may be used by several animal species [2]. Finally, these data cannot be traced to a single farm. Thus, their use in tracking the effectiveness of national antibiotic stewardship programs is severely limited.

Until 2019, data on antibiotic consumption on farms in Italy were available on paper registers. This registration was mandatory for the prescribing veterinarian and under the control of public veterinary services. According to the law [9,10,11] the registrant had to report the following information: (i) date and nature of treatment being prescribed or administered, (ii) identification of treated animals, (iii) suspension time, (iv) identification of the specific drug or feed medication, (v) identification of the drug provider, (vi) medication amount, and (vii) start/end date of treatment. Wherever a stock of medicines was allowed, the registration of used packages and associated stocked quantities on the paper register was also mandatory.

The systematic collection, collation, and analysis of consumption data on Italian farms using paper registers were not feasible at a national level. Therefore, the Italian Ministry of Health launched a new system for the collection of antibiotic consumption data in 2015. The system, which became mandatory in 2019, is based on the direct digitalization of antibiotic sales and the use of antibiotic prescription records [12].

The livestock sector varies greatly in different geographic regions of Italy. In northern Italy, where intensive systems are more common, there exists a population of 3,961,224 beef and dairy cattle (data from the National Livestock Registry, December 31st 2017). In central Italy, the population is reduced to 391,606 cattle, of which 55,468 are in the Umbria region (data from the National Livestock Registry, December 31st 2017). In central and southern Italy, a less intensive livestock system is widespread for both dairy and beef cattle. In cow-calf line and rural dairy farms, farmers are far from implementing a smart livestock system and tradition-bound behaviors are often applied in farm management [13], thereby complicating an effective data collection that should be focused on drug consumption.

According to European priorities and to satisfy consumer demands for welfare-friendly animal-derived products, the Umbria region implemented Measure 14 on animal welfare in the context of the Rural Development Program 2014–2020. The Measure involved 72 dairy farms (a total of 10,284 cows, representing 70% of the total dairy cattle population in the Umbria region) and 268 beef farms (a total of 15,471 cows, representing 41% of the total beef cow population of the region). Actions included in the Measure have been implemented to increase animal welfare, the health status of livestock, farm biosecurity, and drug use through commitments voluntarily chosen by breeders. In a five-year program, farms have been supported both with on-field audits, followed by data collection for assessment, analysis, and monitoring of management practices on the herds. The activities related to Measure 14 and the constant presence of trained technicians in the territory allowed, for the first time in this area, a more detailed data collection in the farms involved, which were chosen to represent the reference population for the present study.

In the sampled farms, paper-based registers were used for the determination of antibiotic consumption. The aim of this study was to provide a qualitative and quantitative characterization of the actual antimicrobial usage recorded on paper-based registers on sample beef and dairy farms located in the Umbria region. The obtained results could represent a stepping stone for further analysis, which will be based on more advanced digital prescription registers.

## 2. Results

### 2.1. Population

**Figure 1:**
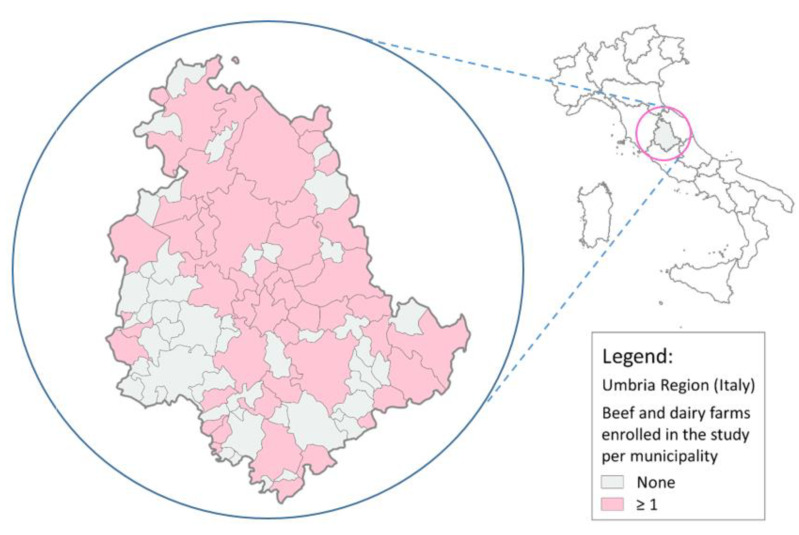
Territorial distribution of sampled farms.

The study involved 101 cattle herds, of which 54 (53.5%) were beef and 47 (46.5%) were dairy (the territorial distribution is shown in Figure 1). The herd size ranged from 51 to 505 head of cattle for beef farms (mean 131 and median 88) and from 51 to 746 for dairy farms (mean 198 and median 132) (for details see Table A1 and Table A2).

The sampled farms represented 4% of the total cattle breeding farms in the Umbria region with at least one animal (101/2496) and 40.9% of those with at least 50 cattle units (101/247). No statistical differences were observed between the sample and the latter regional subpopulation in terms of the distributions of herd sizes on either livestock category (beef: *p* = 0.4806; dairy: *p* = 0.3269). 

The herd size of dairy farms was significantly higher than that of beef farms (*p* = 0.0109).

### 2.2. Number of Treated Animals and Reasons for Being Treated

In terms of the total number of (not necessarily distinct) animals that underwent antibiotic treatment during the observation periods, almost 12,000 antibiotic therapy courses were recorded (2192 and 9742 on beef and dairy farms, respectively).

**Figure 2:**
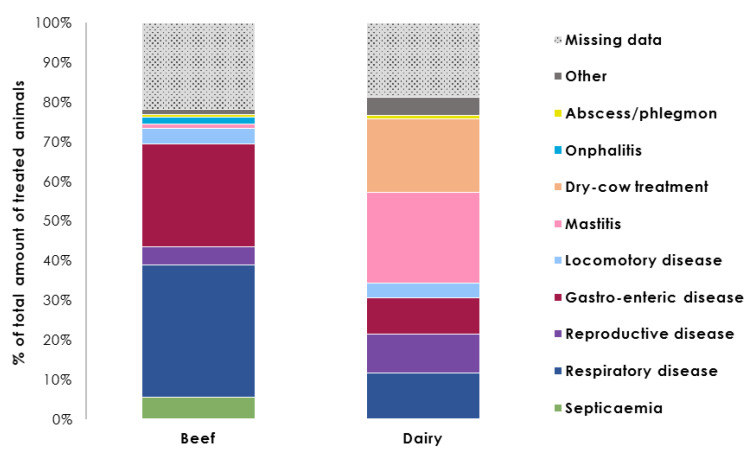
Percentage distribution of treated animals during a yearlong period per individual reason for treatment on beef and dairy cattle farms (for details see Appendix A, Table A3).

The percentage distribution of treated animals per individual reason for treatment on each farm type is shown in Figure 2 (for details see Appendix A, Table A3).

Beef and dairy cattle were treated for different reasons. On beef farms, the principal causes of antibiotic treatments were respiratory (736 treated animals; 33.6%) and gastrointestinal diseases (568; 25.9%); septicemia (118; 5.4%), reproductive diseases (99; 4.5%), and locomotory diseases (85; 3.9%) were less common. Eighty-two percent of treated beef cattle received an injectable drug (1802/2192).

In dairy herds, 41% of antimicrobial therapy cases were related to udder health management; these included mastitis (2222 treated animals; 22.8%) and the dry period (1809 treated animals; 18.6%). Respiratory (1119; 11.5%), reproductive (965; 9.9%), and gastrointestinal diseases (902; 9.3%) were also reported as treatment causes. Fifty percent of dairy cows being treated (4828) were subjected to systemic treatment administration; udder health disorders accounted for 871 of these cases (18%). Treatments to which no reason was assigned accounted for 21.8% on beef farms (478/2192) and 18.8% on dairy farms (1833/9742).

### 2.3. Overall Antibiotic Consumption

**Figure 3:**
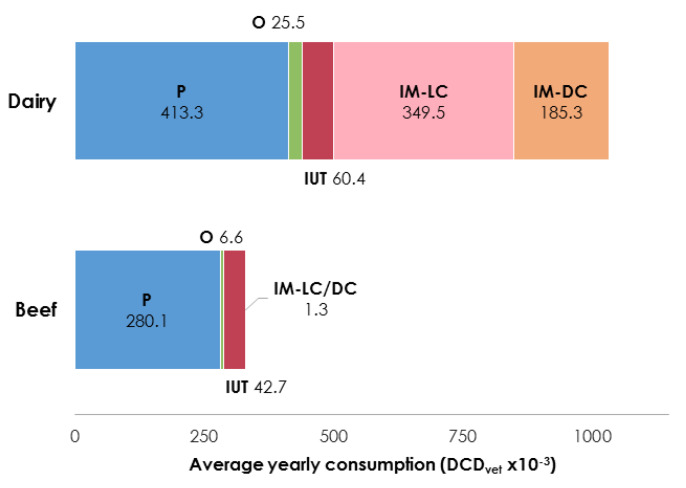
Average yearly consumption (DCD_vet_/year × 10^−3^) per administration route and breeding category (P = parenteral, O = oral, IUT = intrauterine, IM-LC = intramammary lactating cow, IM-DC = intramammary dry cow) (for details see Appendix A, Table A4).

The overall average antibiotic consumption was expressed in defined course doses (DCD_vet_)/year and is presented per livestock specialization and by administration route in Figure 3 (for details see Appendix A, Table A4).

Regarding beef farms, on average 280.1 × 10^−3^ DCD_vet_/year was administered via the parenteral route, representing 85% of the total antibiotic courses administered yearly (330.7 × 10^−3^ DCD_vet_/year). The intrauterine route was the second most popular choice of antibiotic administration (42.7 × 10^−3^ DCD_vet_/year), followed by oral preparations (6.6 × 10^−3^ DCD_vet_/year), and intramammary administration (1.3 × 10^−3^ DCD_vet_/year).

In dairy herds, a mean of 1034.1 × 10^−3^ DCD_vet_/year of antibiotic consumption was registered in total; intramammary treatments accounted for more than 50% of this mean (349.5 × 10^−3^ DCD_vet_/year for lactating cow treatments and 185.3 × 10^−3^ DCD_vet_/year for dry cow treatments). The rest of the antibiotics were administered chiefly systemically (413.3 × 10^−3^ DCD_vet_/year via the parenteral route and 25.5 × 10^−3^ DCD_vet_/year via the oral route), while intrauterine pessaries accounted for 60.4 × 10^−3^ DCD_vet_/year.

**Figure 4:**
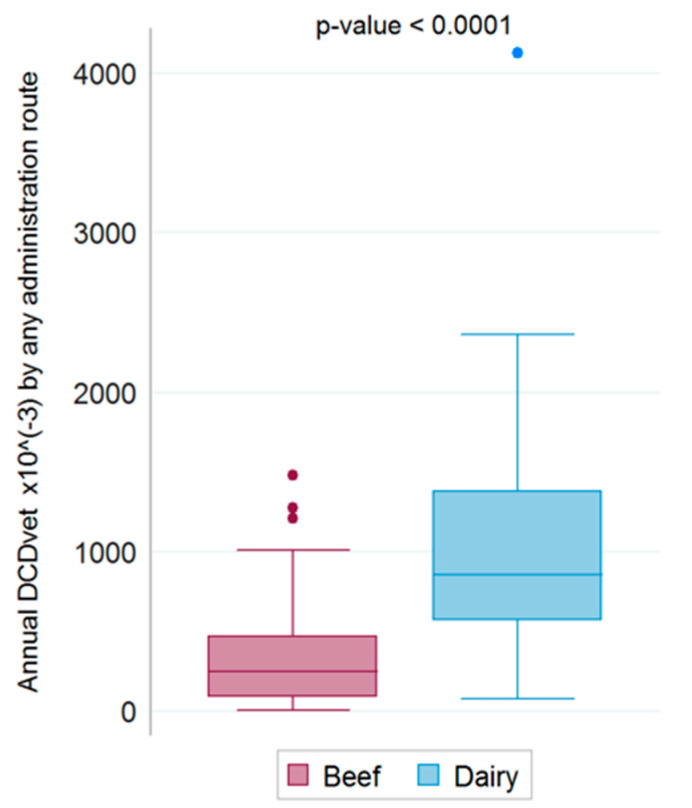
Annual consumption of antibiotics via any administration route on beef and dairy farms.

Boxplots of the total annual antibiotic consumption (DCD_vet_/year) via any administration route on beef and dairy farms are shown in Figure 4.

A Wilcoxon rank sum test revealed a highly significant difference between the two breeding categories in terms of relative overall antimicrobial usage (*p* < 0.0001).

Data on the average consumption via parenteral, oral, and intrauterine administration routes for beef and dairy farms were expressed in defined daily doses (DDD_vet_)/year and DCD_vet_/year and are presented per antimicrobial class in Table 1, Table 2 and Table 3, respectively.

Data concerning the intramammary administration courses were reported for dairy farms only and are presented per combination of antimicrobial agents in Table 4.

Details concerning the distribution of consumptions (DDDvet/year and DCDvet/year) for each administration route, per active ingredient (AI) and antimicrobial class (C) or combination of substances (SC) and combination of classes (CC) are available in Supplementary Materials. In particular, data on consumption via parenteral route, oral route and intrauterine route for each farm are available in Files S1–S3, respectively; data on intramammary administrations courses during the lactating period and the dry period for each farm are provided in Files S4–S5, correspondingly.

### 2.4. Parenteral Route

The results obtained showed that a mean of 756.6 × 10^−3^ DDD_vet_/year was administered via the parenteral route on beef farms. At least one injectable drug was consumed by 52 farms over the course of the observation year (Table 1).

On average, the most used antimicrobial classes on beef cattle were macrolides (178.8 ×10^−3^ DDD_vet_/year), penicillins (157.9 × 10^−3^ DDD_vet_/year), and aminoglycosides (120.3 × 10^−3^ DDD_vet_/year). Penicillins were the first-choice antibiotic class in terms of farms (41/54); apart from these, aminoglycosides and fluoroquinolones were the only two other classes selected by at least half of the sample (Table 1).

In dairy herds, injectable antibiotics accounted, on average, for 1512.9 × 10^−3^ DDD_vet_/year and were used by all the farms (47/47) (Table 1).

In terms of defined daily doses, the most consumed classes on average, were cephalosporins III (455.4 × 10^−3^ DDD_vet_/year), fluoroquinolones (294.5 × 10^−3^ DDD_vet_/year), and penicillins (247.9 × 10^−3^ DDD_vet_/year); moreover, tetracyclines, macrolides, and aminoglycosides accounted for more than 100 × 10^−3^ DDD_vet_/year each (162.3 × 10^−3^, 149.0 × 10^−3^, and 107.6 × 10^−3^ DDD_vet_/year, respectively) (Table 1). On the other hand, only fluoroquinolones and penicillins were administered in over two-thirds of the herds (38 and 36 farms, respectively); these were followed by aminoglycosides, cephalosporins, and macrolides that were consumed by 29, 27, and 25 dairy farms, respectively (Table 1).

The use of polymyxins (colistin) was registered only in few farms (five beef and five dairy farms) and was found only in combination with ampicillin.

**Figure 5:**
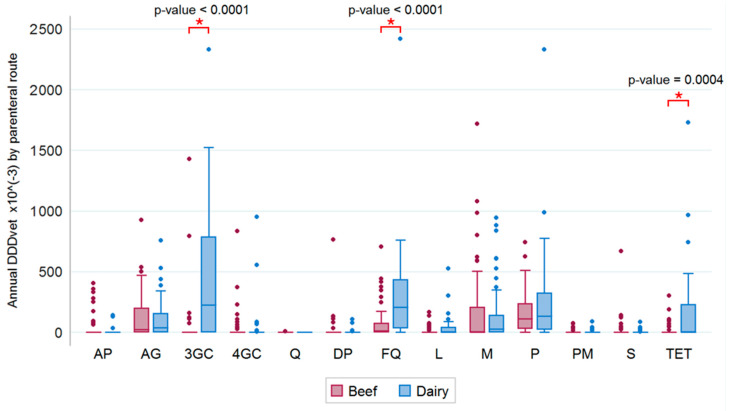
Annual consumption of antibiotics (DDD_vet_/year × 10^−3^) via the parenteral route per antimicrobial class on beef and dairy farms. (AP = amphenicols, AG = aminoglycosides, 3GC/4GC = III/IV generation cephalosporins, Q = quinolones, DP = diaminopyrimidines (trimethoprim), FQ = fluoroquinolones, L = lincosamides, M = macrolides, P = penicillins, PM = polymyxins, S = sulfonamides, TET = tetracyclines).

Boxplots of the annual antibiotic consumption via the parenteral route per individual antibiotic class and with regard to beef and dairy farms are shown in Figure 5. Cephalosporins III, fluoroquinolones, and penicillins were the most highly consumed antibiotics (over 2000 × 10^−3^ DDD_vet_/year).

The results of a Wilcoxon rank sum test showed significant differences between beef and dairy cows regarding the relative antibiotic administration via the parenteral route; this was true for cephalosporins III (*p* < 0.0001), fluoroquinolones (*p* < 0.0001), and tetracyclines (*p* = 0.0004), but not for the other antibiotic classes (Figure 5).

### 2.5. Oral Route

As can be gathered from Figure 3, oral preparations accounted for no more than 2.5% of the total average antibiotic consumption expressed in DCD_vet_/year with respect to either breeding type; herds that registered at least one oral therapy accounted for 18.5% of the beef (10/54) and 32% of the dairy farms (15/47) (Table 2).

Each antibiotic class was not chosen by more than eight to nine farms, however, the most represented classes for beef and dairy farms were sulfonamides, diaminopyrimidines, and aminoglycosides (11.9 ×10^−3^ DDD_vet_/year and 29.2 × 10^−3^ DDD_vet_/year, 9.4 × 10^−3^ DDD_vet_/year and 27.4 × 10^−3^ DDD_vet_/year, and 5.6 × 10^−3^ DDD_vet_/year and 33.4 × 10^−3^ DDD_vet_ /year, respectively) (Table 2).

Oral administrations of polymyxins (colistin) were recorded only on two dairy registers and were used exclusively in combination with ampicillin.

### 2.6. Intrauterine Route

In terms of the total average DCD_vet_/year administered, intrauterine pessaries accounted for 6% and 13% of the treatment courses on beef and dairy farms, respectively (Figure 3), and were used in approximately 30% of both beef and dairy farms.

Rifamycins and tetracyclines were chosen for intrauterine antimicrobial therapies in both farm types. Their average consumption was 57 × 10^−3^ DDD_vet_/year and 28.4 × 10^−3^ DDD_vet_/year, respectively, for beef herds and 103.0 × 10^−3^ DDD_vet_/year and 15.2 × 10^−3^ DDD_vet_/year, respectively, for dairy herds (Table 3).

### 2.7. Intramammary Treatments on Dairy Farms

As anticipated, the use of intramammary products represented over half of the total average antibiotic consumption; 38 dairy farms administered at least one therapy during the lactating period and 43 during the dry period (Table 4).

Lactating cows were primarily given cephalosporins I or penicillins with clavulanic acid (16 and 10 farms, respectively); taking into account all combinations, these classes represented altogether more than half of the total DDD_vet_/year consumed on average (349.5 × 10^−3^ DDD_vet_/year and 263.7 × 10^−3^ DDD_vet_/year, respectively). The mean of cephalosporins IV per se was 235.8 × 10^−3^ DDD_vet_/year and this class had the highest consumption peak.

Dry cow therapies registered on average 185.3 × 10^−3^ DCD_vet_/year, with the most represented classes being cephalosporins I (chosen on 24 farms, 57.5 × 10^−3^ DCD_vet_/year) and penicillins (chosen on 15 farms, 70.5 × 10^−3^ DCD_vet_/year) (Table 4).

### 2.8. Beta-Lactam Consumption

A comparison of the total amount of beta-lactams with other molecules per administration route and breeding type is shown in Table 5. On beef farms, the mean usage of beta-lactams was 66.6 × 10^−3^ DCD_vet_/year, representing 20% of the total average consumption. On dairy farms, almost two-thirds of the total therapy courses contained beta-lactams (mean value 656.4 × 10^−3^ DCD_vet_/year).

### 2.9. Highest Priority Critically Important Antimicrobial (HPCIA) Consumption

According to the WHO classification of molecules with the highest priority [14], the distributions of the average DCD_vet_/year consumed per breeding type, antibiotic category, and administration route are shown in Table 6.

On beef farms, the HPCIAs registered mean of 165.2 × 10^−3^ DCD_vet_/year, were almost exclusively administered via the parenteral route, and accounted for approximately half of the total antibiotic consumption (Table 6).

On the other hand, 36.3% of the total courses carried out on dairy herds consisted of HPCIAs (mean value 375.5 × 10^−3^ DCD_vet_/year); these were mainly injectable drugs (251.9 × 10^−3^ DCD_vet_/year) or intramammary therapies (123.6 × 10^−3^ DCD_vet_/year) (Table 6).

On average, HPCIAs accounted for approximately 50% of the total DCD_vet_/year via non-intramammary routes in both livestock categories.

**Figure 6:**
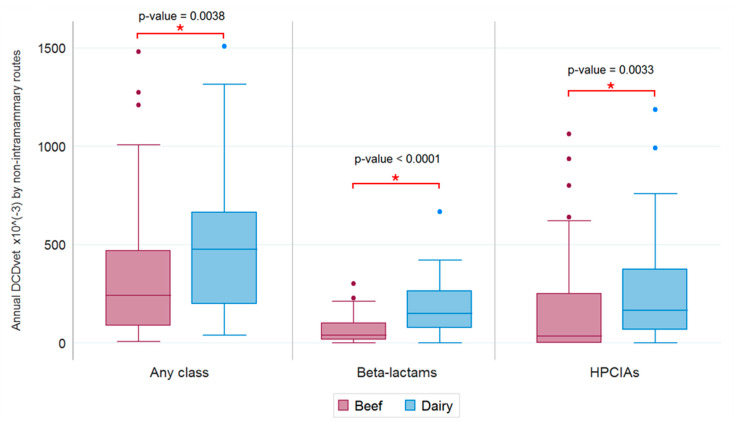
Annual consumption of antibiotic, beta-lactams and HPCIAs, by non-intramammary routes on beef and dairy farms respectively.

Boxplots of the annual consumption of antibiotics, beta-lactams, and HPCIAs, via non-intramammary routes with respect to beef and dairy farms, are shown in Figure 6. The results of a Wilcoxon rank sum test showed a significant difference between beef and dairy farms in terms of the relative overall antimicrobial consumption, (*p* = 0.0038), beta-lactam consumption (*p* < 0.0001), and HPCIA consumption (*p* = 0.0033); these results applied to non-intramammary therapies (Figure 6).

## 3. Discussion

The assessment of antibiotic consumption is the first crucial step in creating an effective plan against AMR. Results obtained by previous studies at a national level [2] should be analyzed in-depth to achieve a permanent reduction in the use of antimicrobials in the context of One Health.

Data collection and analyses can be difficult. Geographical differences, differences in the occurrence of bacterial diseases, farmers’ attitudes, different livestock systems, and the evolution of commercially available products have to be carefully considered. Newly created databases collecting drug prescription, welfare, and production data from Italian farms (Classyfarm®) will allow the analysis of antibiotic consumption at several levels, e.g., for a single animal category or for the cause of treatment. This system is being introduced nationwide in an effort to overcome geographical differences. The data obtained will allow for the comparison of farms and identification of indicators for antibiotics prescription and use. Preventive actions will be implemented, to promote more judicious and responsible antibiotic prescribing and in the long term to decrease AMR of animal pathogens. 

In the veterinary sector, antimicrobial use is often described at a wholesale level [15,16,17]. Data on antimicrobial sales, although useful, do not provide precise information per animal species or per herd and thus have limited value in planning antimicrobial stewardship.

To our knowledge, this is the first study in Italy describing the use of antimicrobials on cattle farms with the use of paper-based registers as the sole source of information.

The choice of collecting antimicrobial consumption data that were self-reported by farmers and/or by on-farm veterinarians involved certain difficulties. Unwritten or illegible information on paper registers was impossible to recover and meant excluding from the study the relative individual treatment being recorded. Additionally, the non-usability of further information on the animals being treated, owing to the heterogeneity of the compilation methods adopted among registers, did not allow analyses at the age category level. Nevertheless, this was an observational study based on already available data, thus offering a retrospective evaluation of antimicrobials actually administered on farms.

As in previous studies [18,19,20], the study population in the present work consisted of a convenience sample based on voluntary participation, therefore it cannot be considered representative of the entire regional population. Additionally, small herds with less than 50 cattle, albeit constituting a large portion of the overall cattle farms in the Umbria region, were not included in the present research. Despite this, the sampled farms appeared to be reasonably characteristic of the regional medium to large size cattle herd subpopulation, thus allowing us to associate the results with a broader scenario.

The absence of a single internationally recognized method to quantify antimicrobial consumption makes direct comparisons with other studies and among different countries or production sectors hard, as has been described in other studies [21,22,23]. The choice of a methodology influences both the numerator (mg, daily doses) and the denominator population (PCU) [24]. Regarding the latter, the estimation of the total mass of liveweight at risk (denominator) depends on the standard liveweight of the adult specimen under consideration, e.g., 500 kg [25,26], 425 kg [24], or 600 kg [20]. The accuracy of this estimation could be related to the inclusion/exclusion of youngstock and different breeds in the analysis. Additionally, differences between metrics could imply substantial differences in the role played by each administration route in the overall antimicrobial consumption. Mass-based metrics (mg/PCU) tend to underscore systemic therapies as the overriding concern to reduce consumption [24]. On the other hand, dose-based metrics (such as DDD_vet_) emphasize the contribution of intramammary therapies, which are calculated in a defined number of injections per cow and not per kg of liveweight. Moreover, even within the intramammary route, the balance can vary between the lactating cow and dry cow treatments (DCTs), depending on the specific dose-based metric chosen. For instance, some authors [27] employed animal-defined daily dosages (ADDD) and counted each tube used for dry cow therapy as one daily dose; this implied a potential overestimation of the DCTs compared with intramammary therapies for lactating cows. On one hand, during lactation, only infected teats are treated; the number of injections per day and the number of days could vary; however, in terms of defined doses, one tube counts as one daily dose (1 DDD_vet_), and three tubes correspond to one therapy cycle (1 DCD_vet_) for a single infected teat. On the other hand, DCTs typically consist of a one-shot treatment for the whole udder (four tubes). Thus, doses per day cannot actually be defined, whereas in terms of complete therapy courses, four tubes used during the dry cow period count as one course. This is the reason why only DCD_vet_ were assigned by the EMA to DCTs. Therefore, DCD_vet_ is the correct metric to use for fair comparisons.

Owing to the lack of the specific DDD_vet_/DCD_vet_ values, the use of some active ingredients (AIs), although observed, was not included in the antimicrobial use calculations. Even though the use of these substances was not significant, an upload of the EMA’s reference tables of DDD_vet_/DCD_vet_ values is recommended in order to include all antimicrobial formulations authorized in Italy.

The following point-by-point discussion of the obtained results provides a complete overview of the quantitative and qualitative analyses performed in this study. Existing literature will be included to compare data, to draw parallels between the dairy and beef sectors at both national and international levels, and to support considerations on the use of beta-lactams and HPCIAs.

### 3.1. Total Amount of Treated Animals and Treatment Reasons

As expected, data on the treatment reasons varied depending on the livestock specialization. In beef farms, as has already been reported by Brault [28], respiratory disease, recognized as the most common cause of mortality [29], was the most commonly treated disease. In dairy herds, according to data reported in Austria [25] and Switzerland [18], udder health problems were the most frequent causes of treatment. In terms of missing data, the higher percentage of treatments to which no reason was assigned that was recorded in beef farms compared with dairy farms, could be explained by comparing the two farming systems. The peculiarity of the cow-calf line system adopted in most of the beef farms included in the study, in which herds are bred in extensive systems for months, often in large pastures, leads to a lower one-by-one check by both farmers and veterinarians. Interestingly, regardless of the livestock specialization, all the farms included in our study used at least once an antimicrobial agent. This result contradicts the results of a German longitudinal study [30], in which a treatment frequency of zero was registered and reported as the median of the treatment frequency per half-year for dairy cows, dairy calves, and beef cattle, in the studied farms. The underlying cause of this discrepancy may be the lower antibiotic use in veterinary medicine registered in Austria compared to Italy, as highlighted by the ESVAC report [8]. Our result may be justified by the composition of our sample, as only farms with at least 50 animals were enrolled in our study; farm consistency is positively associated with antibiotic consumption [30,31].

### 3.2. Parenteral, Oral, and Intrauterine Routes

The parenteral route of administration was used by all dairy farms and the great majority of beef farms; in contrast, the oral and intrauterine routes were used only by 20–30% of the farms.

On beef farms, the absolute number of DDD_vet_ administered parenterally was almost 10 and 30 times greater than the ones registered for the intrauterine and oral routes. On dairy farms, this route of administration was second only to the intramammary route.

Compared to mass medication, the parenteral route is associated with a reduced risk of treating healthy animals and of antibiotic sub-dosing and it is characterized by a rapid onset of action and good bioavailability [32,33]. Parenteral delivery is particularly indicated in cases of gastrointestinal and respiratory disorders, when sick animals reduce their feed and water intake and, therefore, the possible oral assumption of antibiotics [34]. Moreover, the oral administration of antibiotics determines the amplification and development of antibiotic resistance in the gastrointestinal tract, an undesirable effect, which might be minimized using alternative ways of delivery [35].

The popularity of the parenteral route described in our study is not surprising; in the bovine sector, antibiotics are not usually administered via the oral route, either to avoid a disruption of the ruminal microbiota or to prevent drug inactivation by the ruminal flora [32]. Calves are exceptions to this rule, as they are monogastric in the first weeks of their lives [36]. This is in agreement with what has already been reported by Pardon [33], who described how mass medication is rare in cattle, with the exception of veal production, a farming type that was not represented in our sample or in the overall farm population of the Region.

The ranking of parenteral antibiotics showed that penicillins, macrolides, aminoglycosides, and fluoroquinolones were used on the greatest number of farms (Table 1). On the contrary, the oral route was characterized by the use of old antibiotic classes, such as sulfonamides and aminoglycosides. This may well be attributed to the fact that producers may be reluctant to use expensive drugs to treat calves, owing to their low market value. The intrauterine route was characterized by an even narrower spectrum of antibiotics, probably owing to the limited choices of antibiotic preparations dedicated for this use available on the market (https://www.vetinfo.it/j6_prontuario/public/).

The high proportion of farms administering parenteral penicillins is not surprising, as this antibiotic class ranks second for injection preparation in Italy, according to the percentage of sales reported by EMA [8].

In contrast, when the number of parenteral DDD_vet_ was compared in our sample, macrolides and third-generation cephalosporins, two HPCIAs, were the most important antibiotic classes consumed in beef and dairy farms, respectively. This confirms the relevance of parenteral administration in terms of antibiotic consumption.

### 3.3. Intramammary Route on Dairy Farms

The principal administration route for the high percentage of antibiotics prescribed for udder health was the intramammary one. This finding corroborates the results of other studies [20,25,26,37], but it differs from the results of one Canadian and one British study, in which the main route used on dairy farms was the systemic one [19,24].

The results of the present study highlighted that at least one DCT was recorded for almost all dairy farms, however, in terms of DCD_vet_, the intramammary therapies for lactating cows were almost twice as many as those for dry cows. This result is in contrast with what has been reported in other studies, where the amount of antibiotics used for DCT was higher than the one used for intramammary therapy [20,27]; as was explained previously, this discrepancy could be attributed to the different unit of measurement applied. According to Stevens [20], a higher consumption of antibiotics for therapy than for DCT is recorded in high consuming herds. This may explain our results, as the on-farm consumption of antibiotics is generally high in Italy [8]. In this scenario, the prevention of mastitis and antibiotic stewardship aimed at reducing or re-modulating the use of antibiotics for intramammary therapy may represent the most effective strategy to reduce antibiotic consumption in the short term.

The most represented classes were cephalosporins IV and I for lactating cows, while penicillins and cephalosporins I were preferred to cephalosporins IV for DCTs. These results could be compared with those of a study conducted in Pennsylvania where cephalosporins VI and I were the most chosen classes for mastitis, and cephalosporins I and penicillin were most frequently chosen for DCT [31].

The choice of antimicrobial classes for DCT was in line with the suggestions of the national guidelines on the prudent use of first-choice antimicrobials on dairy farms [38]. On the contrary, the choice of cephalosporins IV, as HPCIAs, was in contrast with the aforementioned guidelines.

The correct selection of antibiotics for DCT, aimed at managing subclinical mastitis, could probably be attributed to the minor concern caused compared to lactating cows with clinical symptoms of mastitis where efficacy and withdrawal period influence the choice of antibiotics.

Except for potentiated sulfonamides and rifamycins for lactating cow therapies and DCTs, respectively, the consumption of which was minimal, the remaining used class were beta-lactams. In fact, this was the most represented category of intramammary formulations on the market, considering their half-life, bioavailability, spectrum of action, and suitability to treat intramammary infections.

Macrolides and tetracyclines were not used; these are bacteriostatic antibiotics and are not suggested for intramammary treatments, as not only phagocytosis is reduced in the mammary gland, but also milk interferes with their action [34].

Amphenicols, a first-choice antimicrobial class [38], were not administered, probably because they are less represented in the market of antimicrobial intramammary preparations and just one drug is available in Italy.

### 3.4. HPCIAs

In this study, a high proportion of farms used antibiotics classified as HPCIAs. From 2010 to 2017, there has been a decrease in the overall consumption of antibiotics in the veterinary sector in Italy [8]. However, the same trend was not registered for all molecules and, worryingly, the consumption of many HPCIAs remained steady. As an example, the consumption of third and fourth generation cephalosporins did not vary.

In our study, HPCIAs were generally administered via two routes; the parenteral route was used on both dairy and beef farms and the intramammary route was used only on dairy farms.

The consumption of HPCIAs via the intramammary route in terms of DCD_vet_ on dairy farms was almost the same as the overall HPCIA consumption on beef farms, thereby confirming the importance of antibiotic consumption for udder health.

As stated above, macrolides and third-generation cephalosporins, two HPCIAs, were the most important antibiotic classes administered via the parenteral route in beef and dairy farms, respectively. The use of macrolides is usually associated with the treatment of bovine respiratory disease (BRD) in cattle [28,39]. However, macrolides are included among the third choice drugs for the treatment of respiratory infections in cattle as suggested in the guidelines for the responsible use of antibiotics on dairy farms released by the Emilia-Romagna region in 2017 [38]; thus, macrolides should only be used when other treatment options are not effective, according to culture and in vitro susceptibility testing. The high consumption of third-generation cephalosporins on dairy farms found in this study is in agreement with what has been reported by other authors [20,27]. On beef farms, the consumption of third-generation cephalosporins was lower than on dairy farms (Figure 5); this different attitude to consumption may be justified by the absence of a milk withdrawal period.

In terms of DDD_vet_, fluoroquinolones were used via the parenteral route, ranking second on dairy farms and fourth on beef farms. The use of this antibiotic class is of concern, as it has been discontinued in many European countries in the veterinary sector [27]; according to EMA, the combined sales of fluoroquinolones and other quinolones were <1 mg/PCU in Austria, Denmark, Finland, Iceland, Ireland, Norway, Sweden, Switzerland, and the United Kingdom in from 2010 to 2017 [8]. Colistin, which received great attention by the media in the last four years, was administered parenterally, in conjunction with ampicillin, by approximately 10% of the farms, while only two dairy farms used colistin via the oral route. It should be noted that the use of colistin, alone or in association with another drug, was restricted by the Italian Ministry of Health after an alarming report of a plasmid-mediated mechanism of resistance in late 2015 [40,41]. Consequently, the consumption of polymyxins in the country dropped to 5.2 mg/PCU in 2017 [8].

Overall, a high variation in the parenteral administration of HPCIAs, was observed in our sample, as is shown in Figure 5. For example, one dairy farm consumed 10 times the median amount of fluoroquinolones recorded for farms of the same category. This high variation among herds has already been described by other authors [27] and may be justified by the presence of a specific condition on those farms or by the different attitudes of the prescribing veterinarians. Regardless of the cause, the detection of high-consuming farms has been proposed as a method to reduce antibiotic consumption in the veterinary sector [24]. The data provided here may be pivotal for the establishment of benchmarks for antibiotic consumption on cattle farms in central Italy.

### 3.5. Antibiotic Consumption was Higher on Dairy than on Beef Farms

In our study, we observed that the oral consumption of antibiotics was lower on beef than on dairy farms. It should be noted that on dairy farms, the number of young calves is usually higher than on beef farms. In dairy herds, calves are separated from their mothers and kept in single cages for the first weeks of their lives; this allows frequent health checks and prompt therapy if a disease occurs. By contrast, on beef farms located in the Umbria region, calves are usually nursed by their mothers. These different management practices, which make oral administration easier on dairy farms, may justify our results. The same trend was observed for the parenteral route; the consumption registered on dairy farms was twice as much as that registered on beef farms. The high consumption of antibiotics on dairy farms has been attributed to udder-related problems by some authors [27,30]. In our study, only one-fourth of the antibiotic treatments via the parenteral route were attributed to mastitis, thus we cannot confirm the assumption that udder-related problems were solely responsible for the high consumption observed on dairy farms. This difference could be partially attributed to the type of management characterizing these two farming systems in central Italy. In this area, dairy farms, which raise high-yielding Holstein milk cows, implement remarkably intensive production systems [42], while in beef farms, the dominant cow-calf production system is semi-extensive.

The increased antibiotic consumption trend on dairy farms was further supported by data on beta-lactams and HPCIAs. Overall, the consumption of beta-lactams was 10 times higher on dairy than on beef farms, whereas the consumption of other antibiotics was comparable (Table 5). Even without taking intramammary administration into account, the use of beta-lactams was two times higher on dairy farms than it was on beef farms. The consumption of HPCIAs was more than two times higher on dairy farms than it was on beef farms (Table 5). Even without taking intramammary administration into account, dairy farms still outcompeted beef farms in terms of HPCIA consumption (Table 5). Our results confirm that the intensive management practices adopted by dairy farms are associated with an increased antibiotic consumption. In this scenario, a holistic strategy is required to tackle effectively the antibiotic consumption on bovine farms.

## 4. Materials and Methods

### 4.1. Population

The study population consisted of a sample of cattle farms recruited from September 2017 to April 2018, according to the following criteria: voluntary participation to Measure 14 of the Rural Development Program (Regional)-Umbria 2014–2020 and herd size of at least 50 livestock units.

The sampled farms were categorized according to their management and production target into either dairy or beef farms.

The enrolled farmers signed an informed consent form approving the use of data from their farms for the purposes of this research. The study was conducted in accordance with the Declaration of Helsinki, and the protocol was approved by IZSUM.

### 4.2. Sources of Consumption Data

Data on antimicrobial consumption were extracted from paper-based registers, where according to the law [9,10,11] all therapy courses must be recorded. In order to overcome biases related to the plausible seasonality of antimicrobial consumption [43,44] the observation period was set for each farm as the solar year prior to the herd visit.

The structure of an on-farm paper-based register is described with respect to each treatment in Table 7. Data were digitalized as they were collected and they were organized into a Microsoft Excel spreadsheet (Microsoft Corporation, Redmond, WA, USA) for subsequent analyses.

A lack of data homogeneity and a non-negligible percentage of missing data across different registers led to the exclusion of some variables from the dataset, e.g., the ID/s and the age category of the treated animal/s; unwritten or illegible exact amounts of medication, imposed the exclusion of a few records (i.e., single courses of therapy) from the dataset. Therefore, apart from quantitative details on each antibiotic administration, the information actually employed in this study concerned the number of treated animals and the reason of treatment (Table 7).

### 4.3. Computation of Antimicrobial Use

To provide a rough estimate of the total recorded treatment courses and evaluate the treatment causes over a year-long period within each livestock specialization, the total number of cattle that were subjected to antibiotic treatment was calculated without identifying the animal; thus, an animal was counted as many times as it received at least one antibiotic administration.

Given the considerable number of diagnoses, all reported treatment causes were grouped by the body system involved (e.g., respiratory disease, gastrointestinal disease, etc.) or the relevant category (e.g., DCTs). Less common causes (no more than 2% of the total treated animals per production type) were merged into “others” (see Appendix A, Table A3).

Data on consumption were analyzed using the European Medicine Agency’s dose-based metrics; DDD_vet_ and DCD_vet_ for antimicrobial veterinary medicinal products. These metrics were established within the context of the ESVAC project in order to provide technical units of measurement that take into account dosing substance, and pharmaceutical formulation differences among species [45].

First, each recorded administration was expressed as the number of minimal dosage units (e.g., mL, pill, g, pessary, etc.); then, it was converted into mass of active ingredients (mg) according to the qualitative and quantitative composition of the specific drug.

With respect to the injectable medicines (P) and the oral preparations (O), the number of DDD_vet_ administered yearly per farm was calculated for each AI as follows:

(1) nDDDvet/year(P,O)=total amount of AI (mg)DDDvetvalue assigned to the AI(mgkg×day)×Std.liveweight(kg)×Herd size ,

with 500 kg being considered the standard liveweight [8,25,26]. Taking into account normal fluctuations in the number of animals within a solar year for each farm, the herd size on December 31, 2017 was assumed as reasonably representative of the annual average population at risk, according to the National Livestock Registry.

Analogous were the computations of the nDCD_vet_/year administered via the parenteral or the oral route.

With regard to intramammary products for lactating cows (IM-LC) and intrauterine treatments (IUT), the number of DDD_vet_ administered per year and farm was calculated for each combination of active ingredients (CAI) as follows:

(2) $${\mathrm{nDDD}}_{\mathrm{vet}}/{\mathrm{year}}_{\left(\mathrm{IM}-\mathrm{LC},\mathrm{IUT}\right)}=\frac{\mathrm{total}\;\mathrm{no}.\;\mathrm{of}\;\mathrm{units}\;\left(\mathrm{intramammary}\;\mathrm{tubes}\;\mathrm{or}\;\mathrm{intrauterine}\;\mathrm{devices}\right)}{{\mathrm{DDD}}_{\mathrm{vet}}\mathrm{value}\;\mathrm{assigned}\;\mathrm{to}\;\mathrm{the}\;\mathrm{CAI}\times \mathrm{Herd}\;\mathrm{size}}\;,$$

where the DDD_vet_ value was the defined number of intramammary injections administered daily per teat or the defined number of intrauterine devices administered daily per animal [45]. By employing the DCD_vet_ instead of the DDD_vet_ values, computations of nDCD_vet_/year _(IM-LC, IUT)_ were performed in a similar manner (see Equation (2)).

As no DDD_vet_ values have been allocated to DCTs by the EMA, the analysis of DCTs only included nDCD_vet_/year, with the relative computation being analogous to the nDCD_vet_/year _(IM-LC, IUT)_, with the DCD_vet_ value being the defined number of intramammary injections administered per udder for a therapy cycle.

Italy was not among the nine European Union member states that contributed to the production of these reference values. Owing to the absence of specific DDD_vet_/DCD_vet_ values, the use of some AIs, although observed, was not included in DDD_vet_/DCD_vet_ calculations. Specifically, parenteral therapies containing one of the following AIs were recorded: thiamphenicol, sulfamonomethoxine, dicloxacillin (second AI after ampicillin), sulfamerazine (third AI after sulfadimidine and sulfathiazole); the oral use of procaine benzylpenicillin (second AI after chlortetracycline) was also recorded (for details see Appendix A, Table A5).

Similarly, as no DDD_vet_/ DCD_vet_ values have been assigned by the EMA to topical drugs such as sprays or liquid solutions (uterine lavages), local treatments of dermatitis or reproductive disorders were not included in this analysis (for details see Appendix A, Table A6).

Taking into account the fact that the DDD_vet_ and DCD_vet_ values were assigned at a single substance level for oral and injectable products, while for intramammary and intrauterine products they were assigned by the anatomical therapeutic chemical (ATC_vet_) code (combination of active substances), the antimicrobial use was presented for each administration route by antimicrobial class or by a combination of antimicrobial classes accordingly.

Assessments of overall antimicrobial consumption per livestock category were conducted by nDCD_vet_/year. 

Further specific evaluations were also carried out for beta-lactams and HPCIAs. Beta-lactams included cephalosporins and penicillins with or without clavulanic acid. According to the WHO classification [14], HPCIAs were defined as III/IV generation cephalosporins, quinolones (any generation), macrolides, and polymyxins; with the exception of macrolides, these classes are included in Category B “Restrict” of the most recent categorization of antibiotics for veterinary medicine in the European Union (EU), published by the EMA in December 2019 [17]. This categorization covers all antibiotic classes authorized in human medicine in the EU; four categories were identified depending on the level of risk to public health resulting from veterinary use: A “Avoid”, B “Restrict”, C “Caution”, and D “Prudence”. Category A includes antibiotics prohibited in veterinary medicine, at least with regards to food-producing animals. The classes included in Category B should only be used when there are no alternative antibiotics in a lower category that could be clinically effective; the choice of these restricted classes should be conditional on the result of antibiotic susceptibility testing, whenever possible [17].

### 4.4. Statistical Analyses

Descriptive statistics were performed using Microsoft Excel 2013 (Microsoft Corporation) and Stata^®^ 11.2 statistical software (Special Edition; StataCorp LP, College Station, TX, USA). A map representing the territorial distribution of farms was edited through Qgis 2.18 (https://www.qgis.org/it/site/). Normality of data distributions were checked with the use of the Shapiro-Wilk test. The Student’s *t-*test was used to compare statistically the sizes of the beef and dairy herds. With respect to each livestock category, the same test was performed to compare the herd sizes in the sample with those in the regional subpopulation of cattle farms with at least 50 animals. Then, the Wilcoxon rank sum test was employed in order to assess statistical differences in consumption between the two livestock categories. The significance level was set at α = 0.05. Bivariate statistical analyses were conducted using Stata^®^ (StataCorp LP).

## 5. Conclusions

The results presented and discussed in this paper constitute the first qualitative and quantitative analysis of the actual antimicrobial usage recorded by paper-based registers on sample dairy and beef farms located in the Umbria region, Italy. These results will serve as a stepping stone for further analyses based on more advanced digital prescription registers being developed in Italy and in other countries.

The lack of a single universally recognized standardized method for analyses presented a limitation in the interpretation and discussion of the data. As has already been stated in other papers, shared international standards of data collection and monitoring systems will be crucial in achieving the proposed objectives to reduce AMR worldwide.

An update of the reference tables of the DDD/DCD_vet_ values is recommended, taking into account the molecules authorized in the country level.

The results of this study highlighted how dairy production was associated with a higher risk of antimicrobial use. This is reasonable, taking into consideration the higher fragility of dairy cattle, owing to a shorter productive life and higher specialization. In addition, more intensive management systems that were correlated with herd size in the literature could have been contributing factors. As expected, udder health was confirmed as the principal cause of antibiotic treatment, therefore a crucial factor to manage in order to rationalize antibiotic consumption.

The role of HPCIAs cannot be overlooked, as their use has already been limited or strongly reduced in different countries. Within the parenteral route, the most frequently used method of drug administration, fluoroquinolones represented the second and fourth most frequent antibiotic class used in dairy and beef farms, respectively. Thus, the results of this study highlight the necessity to strengthen the already existing stewardship programs at all levels and involve all potential stakeholders in an effective antimicrobial resistance reduction plan.

## Figures and Tables

**Table 1:** Antimicrobial consumption (DDD_vet_/year × 10^−3^−DCD_vet_/year × 10^−3^) by parenteral route per antimicrobial class and breeding category (for each administration route classes are listed only if they were part of at least one treatment performed on either beef or dairy farms).

PARENTERAL Route—Average Yearly Consumption × 10^−3^ on Beef and Dairy Farms						
Antimicrobial Class	Beef			Dairy		
	No. of Farms	DDD_vet_	DCD_vet_	No. of Farms	DDD_vet_	DCD_vet_
Amphenicols	10/54	38.7	12.6	3/47	6.5	2.1
Aminoglycosides	28/54	120.3	34.2	29/47	107.6	31.5
Cephalosporins III	8/54	54.8	9.7	27/47	455.4	93.2
Cephalosporins IV	11/54	36.5	10.0	10/47	39.5	10.8
Quinolones	1/54	0.1	0.03	0/47	-	-
Fluoroquinolones	29/54	79.2	23.8	38/47	294.5	86.6
Diaminopyrimidines	7/54	25.6	7.2	5/47	4.8	1.3
Lincosamides	13/54	16.5	3.5	18/47	37.0	7.8
Macrolides	19/54	178.8	120.1	25/47	149.0	60.0
Penicillins	41/54	157.9	45.6	36/47	247.9	71.8
Polymyxins	5/54	4.4	1.0	5/47	4.2	0.9
Sulfonamides	10/54	26.0	7.5	7/47	4.3	1.3
Tetracyclines	10/54	17.8	5.0	22/47	162.3	45.9
**Any class**	**52/54**	**756.6**	**280.1**	**47/47**	**1512.9**	**413.3**

**Table 2:** Antimicrobial consumption (DDD_vet_/year × 10^−3^−DCD_vet_/year × 10^−3^) via the oral route per antimicrobial class and breeding category (for each administration route classes are listed only if they were part of at least one treatment performed on either beef or dairy farms).

ORAL Route—Average Yearly Consumption × 10^−3^ on Beef and Dairy Farms						
Antimicrobial Class	Beef			Dairy		
	No. of Farms	DDD_vet_	DCD_vet_	No. of Farms	DDD_vet_	DCD_vet_
Aminoglycosides	6/54	5.6	1.4	9/47	33.4	8.5
Diaminopyrimidines	4/54	9.4	2.0	5/47	27.4	5.7
Macrolides	1/54	0.9	0.7	0/47	-	-
Penicillins	0/54	-	-	3/47	8.4	2.1
Polymyxins	0/54	-	-	2/47	1.7	0.3
Sulfonamides	8/54	11.9	2.5	8/47	29.2	6.0
Tetracyclines	0/54	-	-	2/47	12.1	2.9
**Any class**	**10/54**	**27.8**	**6.6**	**15/47**	**112.2**	**25.5**

**Table 3:** Antimicrobial consumption (DDD_vet_/year × 10^−3^−DCD_vet_/year × 10^−3^) via the intrauterine route per antimicrobial class and breeding category (for each administration route classes are listed only if they were part of at least one treatment performed on either beef or dairy farms).

INTRAUTERINE Route—Average Yearly Consumption × 10^−3^ on Beef and Dairy Farms						
Antimicrobial Class	Beef			Dairy		
	No. of Farms	DDD_vet_	DCD_vet_	No. of Farms	DDD_vet_	DCD_vet_
Cephalosporins I	0/54	-	-	2/47	2.6	1.3
Rifamycins	14/54	57.0	28.5	14/47	103.0	51.5
Tetracyclines	4/54	28.4	14.2	2/47	15.2	7.6
**Any class**	**16/54**	**85.4**	**42.7**	**15/47**	**120.8**	**60.4**

**Table 4:** Annual consumption (DDD_vet_/year ×10^−3^- DCD_vet_/year ×10^−3^) via the intramammary route per combination of antimicrobial agents on dairy farms (single classes or class combinations are listed only if they were part of at least one treatment).

**INTRAMAMMARY LACTATING COW Route—Dairy Farms Yearly Consumption × 10^−3^**					
**Combination of Antimicrobial Agents**	**No. of Dairy Farms**	**DDD_vet_**		**DCD_vet_**	
		**Mean**	**Max**	**Mean**	**Max**
Cephalosporins I	16/47	185.5	2250.0	61.8	750.0
Cephalosporins I + Aminoglycosides	8/47	141.6	3050.8	47.2	1016.9
Cephalosporins I + Rifamycins	3/47	22.3	543.2	7.4	181.1
Cephalosporins III	4/47	28.7	813.2	9.6	271.1
Cephalosporins IV	8/47	235.8	4881.4	78.6	1627.1
Penicillins	8/47	130.5	2757.4	43.5	919.1
Penicillins + Clavulanic Acid	10/47	130.6	1963.6	43.5	654.5
Penicillins + Aminoglycosides	1/47	2.6	121.2	0.9	40.4
Sulfonamides + Trimethoprim	7/47	171.0	2930.5	57.0	976.8
**Any association**	**38/47**	**1048.6**	**8915.3**	**349.5**	**2971.8**
**INTRAMAMMARY DRY COW Route—Dairy Farms Yearly Consumption × 10^−3^**					
**Combination of Antimicrobial Agents**	**No. of Dairy Farms**	**DCD_vet_**			
		**Mean**		**Max**	
Cephalosporins I	24/47	57.5		336.2	
Cephalosporins IV	8/47	35.5		508.5	
Penicillins	15/47	70.5		444.8	
Penicillins + Aminoglycosides	10/47	21.1		214.5	
Rifamycins	1/47	0.7		33.3	
**Any association**	**43/47**	**185.3**		**508.5**	

**Table 5:** Average yearly consumption of beta-lactams (DCD_vet_/year × 10^−3^) per individual administration route on beef and dairy farms (P = parenteral, O = oral, IUT = intrauterine, IM-LC = intramammary lactating cow, IM-DC = intramammary dry cow).

Average Yearly Consumption on Beef and Dairy Farms (DCD_vet_/year × 10^−3^)									
Livestock Category	Antimicrobial Classes Category	Administration Route							Overall Total
		P	O	IUT	Total	IM-LC	IM-DC	Total	
Beef	Beta-lactams *	65.3	-	-	65.3	0.7	0.6	1.3	66.6
	non-beta-lactams	214.8	6.6	42.7	264.1	-	-	-	264.1
Dairy	Beta-lactams *	175.8	2.1	1.3	179.2	292.5	184.6	477.2	656.4
	non-beta-lactams	237.5	23.4	59.1	320.0	57.0	0.7	57.7	377.7

* including combinations of antimicrobial agents containing at least one beta-lactam (this applied for intramammary routes).

**Table 6:** Average yearly consumption of HPCIAs (DCD_vet_/year × 10^−3^) per individual administration route on beef and dairy farms. P = parenteral, O = oral, IUT = intrauterine, IM-LC = intramammary lactating cow, IM-DC = intramammary dry cow.

Average Yearly Consumption on Beef and Dairy Farms (DCD_vet_/year × 10^−3^)									
Livestock Category	Antimicrobial Classes Category	Administration Route							Overall Total
		P	O	IUT	Total	IM-LC	IM-DC	Total	
Beef	HPCIAs *	164.5	0.7	-	165.2	-	-	-	165.2
	non-HPCIAs	115.6	5.9	42.7	164.2	0.7	0.6	1.3	165.5
Dairy	HPCIAs *	251.5	0.3	-	251.9	88.2	35.5	123.6	375.5
	non-HPCIAs	161.8	25.2	60.4	247.3	261.4	149.9	411.3	658.6

* including combinations of antimicrobial agents containing at least one HPCIAs (this applied to intramammary routes).

**Table 7:** Structure of an on-farm paper-based register (with respect to each treatment), and information selected for antimicrobial consumption analysis.

Data Field	Explanation	Selected
Type of treatment register	Unified register (drugs stocks plus treatments records) or treatment register	✓
Veterinarian ID	Refers to the veterinarian who is responsible of prescription	
Farm ID	Identifies each individual farm	✓
Farm production type	e.g., dairy, beef	✓
Animal species	Cattle	
Loading/unloading date	(only if stocks are allowed)	
Medicine packages loaded/unloaded	(only if stocks are allowed)	
Prescription date		
Commercial name of the drug		✓
Medication lot number		
Reason of treatment	e.g., pneumonia, mastitis	✓
Dosage	e.g., 6 mL on 3–4 consecutive days	
Treatment start date		✓
Treatment end date		
Medication amount	e.g., 20, 1, 4, etc.	✓
Medication unit	e.g., mL, litre, g, tube, pessary, etc.	✓
Medication residual amount		
No. of treated animals		✓
Treated animal/s ID/s	e.g., ear tag number, box number, identification code, etc.	
Animal/s sex		
Animal/s age category	e.g., calf, heifer, bullock, etc.	
Length of treatment	In days	
Statutory withdrawal period (milk/meat)	In days	

## Supplementary Materials

The following are available online at https://www.mdpi.com/2079-6382/9/5/273/s1. File S1: Data on antimicrobial consumption via parenteral route (DDD_vet_/year-DCD_vet_/year) per farm, active ingredient (AI), and antimicrobial class (C). File S2: Data on antimicrobial consumption via oral route (DDD_vet_/year-DCD_vet_/year) per farm, active ingredient (AI), and antimicrobial class (C). File S3: Data on antimicrobial consumption via intrauterine route (DDD_vet_/year-DCD_vet_/year) per farm, combination of substances (SC), and combination of antimicrobial classes (CC). File S4: Data on antimicrobial consumption via intramammary route for lactating cows (DDD_vet_/year-DCD_vet_/year) per farm, combination of substances (SC), and combination of antimicrobial classes (CC). File S5: Data on antimicrobial consumption via intramammary route during dry period (DCD_vet_/year) per farm, combination of substances (SC), and combination of antimicrobial classes (CC). In Supplementary Materials, active ingredients (AI), antimicrobial classes (C), and combinations of substances (SC) are identified according to EMA abbreviations [45].

## Appendix A

**Table A1:** Sampled farms distribution per livestock category and herd size.

Herd Size (Head of Cattle)	No. of Farms		Total
	Beef	Dairy	
50–99	29	20	49
100–499	24	23	47
≥500	1	4	5
Total	54	47	101

**Table A2:** No. of farms and farm size (mean, median, minimum, and max) per breeding type in the sampled farms and in Umbria Region in the same period.

Herds			Herd Size			
Type		No.	Mean	Median	Min.	Max
Beef	Sampled	54	131	88	51	505
	Umbria Region	174	119	82	50	895
Dairy	Sampled	47	198	132	51	746
	Umbria Region	73	167	104	51	746

**Table A3:** Total no. of treated animals during the study period, per livestock category and reason of treatment.

No. of Treated Animals Per Reason of Treatment on Beef and Dairy Farms				
Reason of Treatment	Beef (tot. 2192)		Diary (tot. 9742)	
	No.	%	No.	%
Septicaemia	118	5.4%	0	0%
Respiratory disease	736	33.6%	1119	11.5%
Reproductive disease	99	4.5%	965	9.9%
Gastro-enteric disease	568	25.9%	902	9.3%
Locomotory disease	85	3.9%	355	3.6%
Mastitis	26	1.2%	2222	22.8%
Dry-cow treatment	1	0.05%	1809	18.6%
Onphalitis	35	1.6%	1	0.01%
Abscess/phlegmon	16	0.7%	91	0.9%
Other ^a,b^	30 ^a^	1.4%	445 ^b^	4.6%
Missing data	478	21.8%	1833	18.8%

^a^ including fever, wound/injury, post-surgical prophylactic treatment, rheumatism; ^b^ including dermatitis, fever, post-surgical prophylactic treatment, nephritis, ketosis, foreign body syndrome, mycoplasma otitis, chlamydiosis, reticuloperitonitis.

**Table A4:** Average yearly consumption (DCD_vet_/year ×10^−3^) per individual administration route on beef and dairy farms respectively (P = parenteral, O = oral, IUT = intrauterine, IM-LC = intramammary lactating cow, IM-DC = intramammary dry cow).

Average Yearly Consumption on Beef and Dairy Farms (DCD_vet_/year × 10^−3^)								
Livestock Category	Administration Route							Overall Total
	P	O	IUT	Total	IM-LC	IM-DC	Total	
Beef	280.1	6.6	42.7	329.4	0.7	0.6	1.3	330.7
Dairy	413.3	25.5	60.4	499.2	349.5	185.3	534.9	1034.1

**Table A5:** Active ingredients devoid of DDD_vet_/DCD_vet_ values and relative usage.

AIs Devoid of DDD_vet_/DCD_vet_ values	Association	Administration Route	No. of Farms	No. of Treated Animals
*Thiamphenicol*	*-*	Parenteral	1 (dairy)	4
*Sulfamonomethoxin*	*Trimethoprim*	Parenteral	1 (dairy)	10
*Dicloxacillin*	*Ampicillin*	Parenteral	22 (dairy)	244
*Sulfamerazine*	*Sulfadimidine + Sulfathiazole*	Parenteral	2 (beef)	34
*Procaine benzylpenicillin*	*Chlortetracycline*	Oral	1 (dairy)	1

**Table A6:** Topical treatments excluded from DDD_vet_/DCD_vet_ calculations (AI = active ingredient).

AIs	Usage	No. of Farms	No. of Treated Animals
*Oxytetracycline*	Uterine lavage	2 (beef)	2
*Oxytetracycline*	Uterine lavage	5 (dairy)	221
*Rifaximin*	Intrauterine foam	1 (dairy)	1
*Oxytetracycline*	Local treatment of dermatitis	1 (dairy)	4
